# Failure of Interferon γ to Induce the Anti-Inflammatory Interleukin 18 Binding Protein in Familial Hemophagocytosis

**DOI:** 10.1371/journal.pone.0008663

**Published:** 2010-01-13

**Authors:** Claudia A. Nold-Petry, Thomas Lehrnbecher, Andrea Jarisch, Dirk Schwabe, Josef M. Pfeilschifter, Heiko Muhl, Marcel F. Nold

**Affiliations:** 1 Pharmazentrum Frankfurt, Johann Wolfgang Goethe-University Hospital, Frankfurt, Germany; 2 Department of Pediatrics, Johann Wolfgang Goethe-University Hospital, Frankfurt, Germany; 3 Division of Infectious Diseases, University of Colorado Denver, Aurora, Colorado, United States of America; University of California San Francisco, United States of America

## Abstract

**Background:**

Familial hemophagocytosis (FHL) is a rare disease associated with defects in proteins involved in CD8^+^ T-cell cytotoxicity. Hyperactivation of immune cells results in a perilous, Th1-driven cytokine storm. We set out to explore the regulation of cytokines in an FHL patient who was clinically stable on low-dose immunosuppressive therapy after bone marrow transplantation over a six-month period. During this period, chimerism analyses showed that the fraction of host cells was between 1 and 10%. Both parents of the patient as well as healthy volunteers were studied for comparison.

**Methods/Principal Findings:**

Using ELISA, quantitative real-time PCR, and clinical laboratory methods, we investigated constitutive and inducible cytokines, polymorphisms, and clinical parameters in whole blood and whole blood cultures. Although routine laboratory tests were within the normal range, the chemokines IP-10 and IL-8 as well as the cytokine IL-27p28 were increased up to 10-fold under constitutive and stimulated conditions compared to healthy controls. Moreover, high levels of IFNγ and TNFα were produced upon stimulation. Unexpectedly, IFNγ induction of IL-18 binding protein (IL-18BP) was markedly reduced (1.6-fold vs 5-fold in controls). The patient's mother featured intermediately increased cytokine levels, whereas levels in the father were similar to those in the controls.

**Conclusions/Significance:**

Since IL-18 plays a major role in perpetuating hemophagocytosis, the failure of IFNγ to induce IL-18BP may constitute a fundamental pathogenetic mechanism. Furthermore, increased production of IL-8 and IL-27 appears to be associated with this disease. Such dysregulation of cytokines was also found in the heterozygous parents, providing a novel insight into genotype-phenotype correlation of FHL which may encourage future research of this rare disease.

## Introduction

Hemophagocytic lymphohistiocytosis (HLH), also called hemophagocytic or macrophage activation syndrome, is the name of a group of rare diseases characterized by a dysregulation of the immune system. HLH can be inherited, but occurs more commonly secondary to causes such as viral (including H5N1) or bacterial infections or cancer. The group of inherited HLH comprises familial HLH (FHL) and the Chédiak-Higashi and Griscelli syndromes, which are associated with various gene defects, but are nearly identical in clinical presentation. Clinical features, which are shared by the inherited and the acquired forms, include nonremitting high fever, hepatosplenomegaly, cytopenia of at least two lineages, and/or elevations in pro-inflammatory cytokines and soluble CD25. In severe cases, multi-organ failure may ensue. Phagocytosis of hematopoetic cells in the bone marrow, spleen, or lymph nodes caused by dysregulation of T-cells, NK cells, and macrophages is characteristic of HLH. FHL usually is manifest in infancy with fulminant failure of several organs and, without chemo- and immunotherapy followed by bone marrow transplantation (BMT), is almost always fatal.

Since FHL is rare with 240 reported cases between the first description by Farquhar and Claireaux in 1952 [1] and 1997 [2], investigative opportunities are limited. The association of mutations in the perforin gene with the disease [3] was a groundbreaking discovery; however, in some patients with acute FHL, the perforin gene is intact whereas in others the disease presents late or not at all despite a mutation [4]. To date, five mutations in genes coding for proteins involved in cytotoxicity of lymphocytes, namely perforin (causing FHL2), Munc13–4 (FHL3), syntaxin-11 (FHL4) [5], and STXBP2 (FHL5) [6] have been found to cause FHL. The gene responsible for FHL1 remains to be identified. FHL2 to 4 account for about 80% of FHL cases. Recent studies have further advanced the understanding of the link between the perforin defect and the cytokine storm of acute HLH, the latter of which ultimately causes the rapidly progressive multi-organ failure. Several cytokines, including IL-6, TNFα [7], IL-1β [8], IL-12 [9], and IL-18 [10]–[13] are produced at markedly high levels during the acute phase of the disease. However, more recent reports have emphasized the particular importance of IFNγ and the subsequent Th1 activation in the pathogenesis of the disease [10]–[14]. In fact, levels of free IL-18 are not only elevated [11], but also correlate with disease severity [13] and may account for increased expression of IFNγ and FasL, as well as for liver inflammation. High levels of IFNγ, which was previously called macrophage activating factor, may contribute to bone marrow suppression in HLH.

We therefore investigated parameters of Th1- and general immune activation in an FHL patient with a perforin mutation. She was studied for 6 months starting 7 months after BMT during which a mixed chimerism (1–10% host) was present. We compared the data to the patient's heterozygous parents and a collective of healthy individuals (HC). In addition to demonstrating dysregulation of several cytokines, we observed that the induction of IL-18 binding protein (IL-18BP), the only known natural inhibitor of IFNγ and Th1 responses in humans [10], [15], was impaired in the FHL patient's blood cells.

## Materials and Methods

### Ethics Statement

Informed written consent was obtained from the patient's mother and father as well as from all HC. The experiments were approved by the Ethikkomission of the J.W.Goethe University and conducted in compliance with the Helsinki Declaration.

### History

At 3 months of age, the female infant was admitted to the pediatric intensive care unit of the J. W. Goethe University Hospital at Frankfurt am Main with the classic presentation of FHL characterized by a sepsis-like clinical picture with hepatosplenomegaly, pancytopenia, and impaired liver function. FHL was confirmed by bone marrow histology and FACS analysis (perforin absent on lymphocytes, G149S mutation). The consanguineous parents (first degree cousins) are heterozygotes.

Chemotherapy according to HLH94 [16], [17], including etopophos, cyclosporine A, dexamethasone, and intrathecal methotrexate was initiated 4 days after admission and the initial response was good. At 6 months of age, the patient underwent BMT using marrow from an HLA (high resolution)-identical donor. Hematopoetic recovery was adequate. Post-BMT complications included one episode of graft-versus-host disease of the skin on days 20 to 25, which quickly remitted under short-term prednisolone.

The patient was in good overall condition and chimerisms were between 1 and 10% host cells during study period. Informed written consent was obtained from the patient's mother and father as well as from all HC. All experiments were conducted in compliance with the Helsinki Declaration.

### Reagents

Chemicals, reagents and kits were obtained from the following companies: E. coli-LPS (O127∶B8): Sigma, Munich; IFNγ: Peprotech, Frankfurt; RPMI 1640 medium and FCS: Life Technologies GibcoBRL, Karlsruhe; IL-8 and IP-10 ELISA kits: BD Pharmingen, Heidelberg; all Germany. PCR reagents and devices: Applied Biosystems, Foster City, CA, USA.

### Whole Blood (WB) Culture

Equal volumes of freshly obtained heparinized blood were mixed with RPMI1640 plus 25 mM Hepes, 100 U/ml penicillin, and 100 µg/ml streptomycin. 1 ml aliquots were then transferred into round-bottom polypropylene tubes which were incubated at 37°C and 5% CO_2_ for 20 h.

### Detection of Cytokines

WB cultures were mixed, then one aliquot was lysed with Triton X-100 (final concentration 1%) for determination of IL-8. The other aliquot was centrifuged at 350 g and the cell-free supernatant was removed and assayed for IP-10. ELISAs were performed according to the manufacturer's instructions.

### Isolation of RNA and PCR

Total RNA from WB cultures was obtained using the QIAamp RNA Blood Mini Kit from Qiagen (Hilden, Germany). Primers and probe for IL-18BPa real-time PCR were as follows: primers, 5′-acctcccaggccgactg-3′ and 5′-ccttgcacagctgcgtacc-3′; probe, 5′-caccagccgggaacgtggga-3′ (exon/intron spanning). For GAPDH, pre-developed assay reagents (Applied Biosystems) were used. Specificity of PCR products was tested by classic PCR using the aforementioned primers. mRNA copy numbers for IL-18BPa and GAPDH were determined using cloned cDNA standards. For analysis of IL-27p28, 35 cycles of a PCR with the primers 5′-gcggaatctcacctgccag-3′ and 5′-cgggaggttgaatcctgca-3′ were run.

### Cytokine Promoter Analysis

Variant alleles of the cytokine promoters IL-6 (G-174-C), IL-8 (A252T), and TNFα (G-308A) were performed as described in detail previously [18], [19].

### Statistical Analysis

Data were analyzed by Student's t-test.

## Results

### Elevated Cytokine Levels in WB of Family Members

Chimerism studies indicated that during the time of these investigations, 1 to 10% of blood cells were of host origin. The ability of the patient's whole blood to produce Th1 and effector cyto-/chemokines under steady-state as well as stimulated conditions was compared to that in phenotypically healthy heterozygotes, namely both parents, as well as to that in HC.

**Figure 1:**
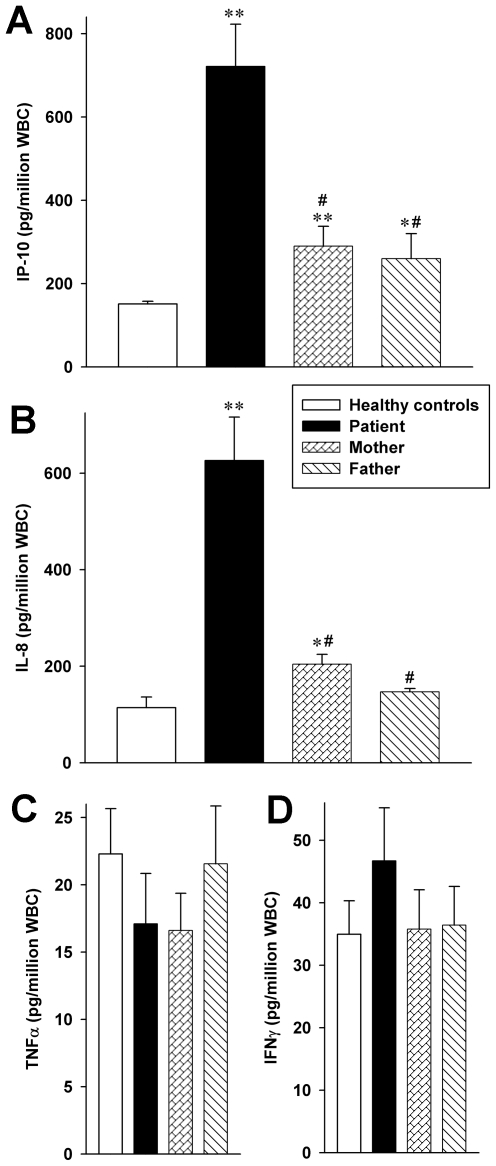
Excessive cytokine production in WB cultures from the FHL patient. WB culture were incubated for 20 h; thereafter, ELISA was performed on lysates (IL-8) or supernatants (others). Data are shown as cytokine protein in pg normalized to 1 million white blood cells ± SEM, n = 4 time points for IP-10 and IL-8 and n = 3 for IFNγ and TNFα spanning 6 months for all subjects; the same three healthy donors were tested on each time point. *, p<0.05 and **, p<0.01 for healthy controls vs patient, mother, and father; #, p<0.05 and ##, p<0.01 for patient vs mother and father.

As shown in **Figure 1A and B**, the average basal production of IP-10 and IL-8 during the study period was markedly increased in the FHL patient compared to HC. Both chemokines were also significantly elevated in the patient's mother; IP-10 was increased in the father. In contrast, constitutive production of IFNγ and TNFα was similar in each subject (panels **C** and **D**).

**Figure 2:**
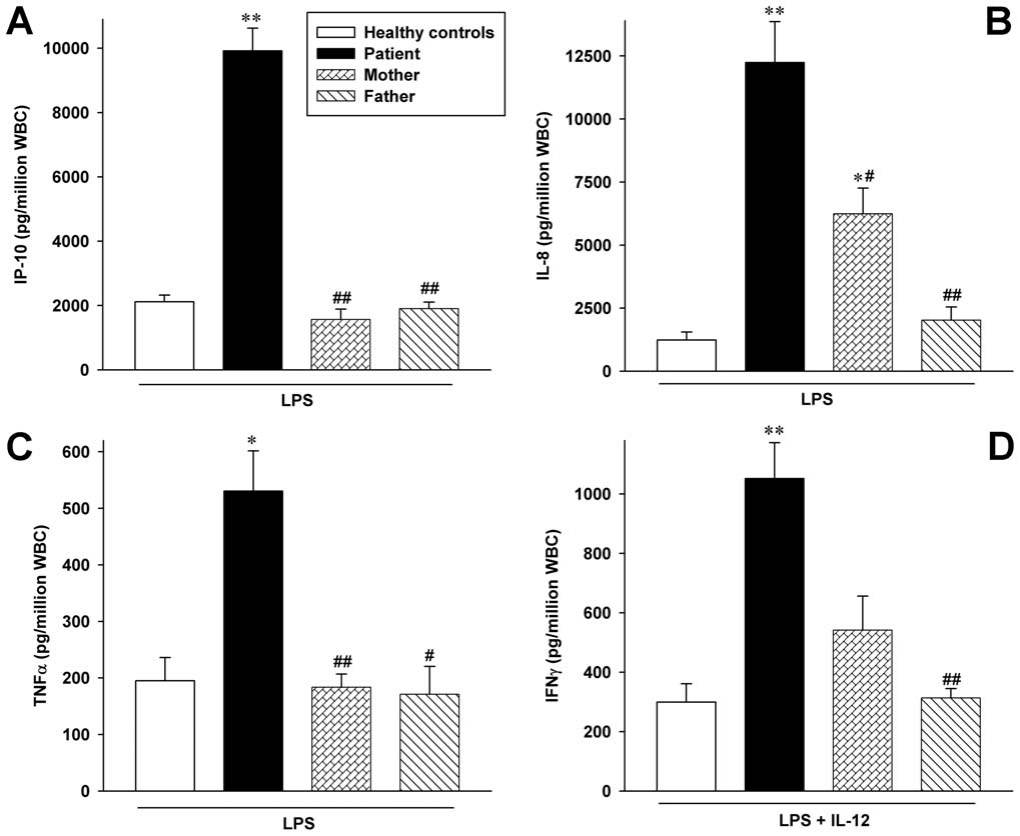
Cytokine levels in WB cultures stimulated with LPS. WB cultures were incubated in the presence of 100 ng/ml LPS for 20 h. In panel **D**, IL-12 (20 ng/ml) was also added. IL-8 was measured in cell lysates, IP-10, TNFα, and IFNγ were determined in the supernatants. Data are depicted as cytokine protein in pg normalized to 1 million white blood cells ± SEM, n = 4 time points for IP-10 and IL-8 and n = 3 for IFNγ and TNFα over a period of 6 months for all subjects; the identical healthy volunteers were tested on each time point. *, p<0.05 and **, p<0.01 for healthy controls vs patient, mother, and father; #, p<0.05 and ##, p<0.01 for patient vs mother and father.

Upon stimulation with LPS, WB cultures from mother, father, and HC secreted comparable amounts of IP-10, TNFα, and IFNγ protein (**Figure 2**), whereas the patient produced considerably more IP-10 (5-fold), TNFα, and IFNγ (both 3-fold). IL-8 release after LPS in the patient was more than 10-fold higher than in HC. The mother also produced significantly more IL-8 than father and controls. She featured a similar pattern for IFNγ when IL-12 was used as an additional stimulus (panel **D**).

In family members and in HC, constitutive levels of IL-6 were below detection limits (5 pg/ml) and measurements of CRP, white blood count including differential IgG, IgA, and IgM, LDH, and kidney function were each within normal limits throughout the study period. Only parameters of liver function, namely ALT, AST, and γGT, were slightly elevated in the patient.

### Cytokine Promoter Polymorphism Analysis

Identical genotypes of the promoters of the IL-8 and TNFα genes were found in the mother, father, and the patient (heterozygous AT for IL-8, homozygous for allele A for TNFα). The same was the case for FcγRIIa and IIIb. For IL-6, the father was heterozygous, whereas both patient and mother were homozygous for allele G, which is associated with a higher production of the cytokine [20].

### Dysregulation of Anti-Inflammatory IL-18BP and IL-27p28

**Figure 3:**
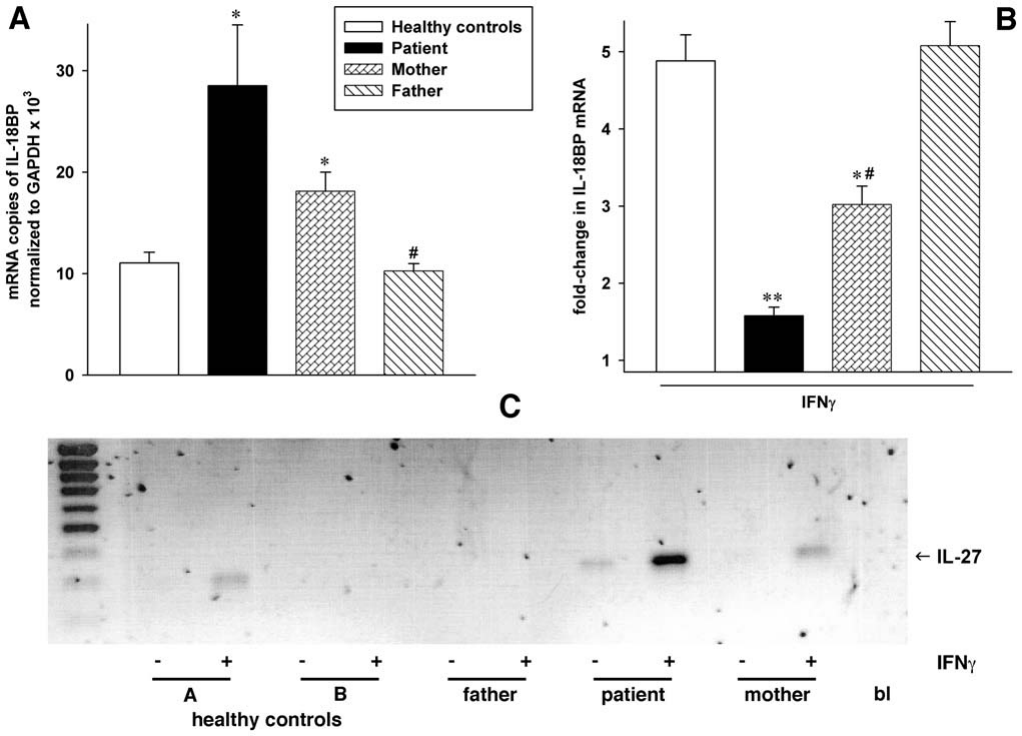
Regulation of IL-18BP and IL-27p28. RNA was isolated from WB culture after a 20 h incubation with or without 50 ng/ml IFNγ. **A,** Data are shown as absolute copy numbers of IL-18BP normalized to GAPDH×10^3^± SEM, n = 4 time points for all subjects; three control individuals were assayed on each time point. **B,** IFNγ-induced fold-changes in normalized IL-18BP mRNA copy numbers are shown. **A** and **B,** *, p<0.05 and **, p<0.01 for healthy donors vs patient, mother, and father; #, p<0.05 for patient vs mother and father. **C,** IL-27p28 PCR from the same samples. Differences in GAPDH copy numbers were negligible. One representative of 3 different gels showing similar results is depicted. Healthy donor C exhibited no expression of IL-27p28 (not shown).

Despite immunosuppressive therapy, markedly increased levels of several pro-inflammatory cytokines were produced in WB cultures from the patient. We next investigated two anti-inflammatory mediators, IL-18BP and IL-27. It is known that IFNγ is a potent inducer of IL-18BP in vitro [21] as well as in patients treated with IFNα [22]. Although steady-state expression of IL-18BP in whole blood was moderately elevated in the patient (**Figure 3A**), the induction of IL-18BP by IFNγ was greatly reduced compared to HC (1.6-fold vs 5-fold, panel **B**). Whereas IL-18BP expression in the father's cells was nearly identical to that of the controls, the cells of the mother exhibited intermediate steady-state and IFNγ-induction patterns. In contrast to IL-18BP, steady-state expression as well as IFNγ-induction of IL-27p28 was markedly higher in the patient than in the parents and in HC (**Figure 3C**).

## Discussion

Previous studies have revealed a pivotal role for Th1 cytokines in patients with FHL. For instance, antibodies to IFNγ, but not to other cytokines, improved the course of the disease including survival in an animal model of FHL [14]. In patients, IL-18 [11]–[13] and IFNγ [13] correlate with disease severity. Although both IL-18BP and IP-10 were elevated in WB cultures from our patient under steady-state conditions, the near absent induction of the Th1 antagonist IL-18BP by IFNγ was striking. In contrast, LPS-induced increases in the pro-inflammatory chemokines IP-10 (14-fold) and IL-8 (20-fold) were comparable to or even greater than in control subjects. This suggests the conclusion that, despite BMT and immunosuppression, regulation of IL-18BP is severely impaired in the FHL patient. The induction of IL-18BP by interferons, demonstrated by Paulukat et al in vitro [21], by Kaser et al in vivo [22], as well as the characterization of the IFNγ-responsive promoter [23], [24] each constitute a negative-feedback loop to reduce IFN activity and the subsequent Th1 responses [10]. Hence, our data appear to reveal a fundamental pathophysiological mechanism of HLH, that is, escape of IL-18BP from IFNγ stimulation. Consistent with this concept is the observation that in secondary HLH, low levels of IL-18BP result in high free IL-18 [11]. Of note, IL-18BP also reduces inflammation caused by IL-18 directly, i.e. independent of IFNγ. This fact should be taken into account when the results of two other studies are contemplated, in which a dysregulation of the IL-18/IL-18BP equilibrium in systemic lupus erythematosus (SLE) [25] and Wegener's granulomatosis (WG) [26] was reported. In fact, analysis of the responsiveness of IL-18BP production to IFN stimulation may be a worthwhile topic of research in SLE and WG.

Our patient was in good general condition and most laboratory parameters were normal, but she featured an incomplete chimerism and slightly elevated liver enzymes. These clinical findings were accompanied by a pro-Th1 twist of steady-state and inducible cytokine levels. Unexpectedly, cytokine production abnormalities were also detectable both healthy parents, with the mother resembling the patient at a smaller scale, but the father showing only slight abnormalities. Individuals heterozygous for perforin mutations have previously been studied regarding the activity of their NK cells [4], [27], [28], but changes in cytokine levels have not been reported before. Although the molecular mechanisms for the differences in the parent's cytokine regulation patterns remain unclear, these differences are in accord with varying degrees of NK cell function in heterozygotes [27], [28]. Taken together, the findings in the patient (which likely indicated a smoldering disease state) and in the parents establish a novel vantage point from which the genotype-phenotype correlations of FHL can be investigated in future studies.

Based on data in perforin-deficient mice [14], the pathophysiology of FHL might include presentation of a foreign (e.g. viral) antigen which results in recruitment and stimulation of cytotoxic CD8^+^ T-cells. Besides attempting to kill infected cells via perforin, activated CD8^+^ T-cells secrete cytokines, including IFNγ. Since CD8^+^ T-cells from patients with perforin-associated FHL fail to kill infected cells, these killer cells continue to activate macrophages and to deliver proliferation signals to T-cells. In turn, T-cells and macrophages perpetuate the production of mostly Th1 and pro-inflammatory cytokines and, in the case of macrophages, uncontrolled phagocytosis is initiated [29]. However, it is important to bear in mind that this animal model has its limitations: a) the observations are based on infection with one virus; a different infectious agent may produce different results. b) Not every case of human FHL/HLH is caused by a perforin mutation - defects in other proteins can elicit a similar clinical picture although there may be differences in pathophysiology. c) It appears unlikely that CD8^+^ T-cell-derived IFNγ is the only factor which drives the deleterious inflammation in humans. IFNγ may be produced by other cells and, most importantly, macrophages likely play a more prominent role, since IL-18 is closely related to disease severity in human FHL/HLH [11]–[13]. Macrophage-derived IL-18 may in fact bypass IFNγ, directly causing inflammation. These properties of IL-18 may help explain why this cytokine appears more critical than others, e.g. IL-12 [13], to the pathogenesis of FHL/HLH and highlight the importance of the dysregulation of IL-18BP for the disease process. We suggest that IL-18BP deserves consideration as a therapeutic agent in diseases in which a dysregulation of the IL-18/IL-18BP equilibrium is evident. This is the case in SLE [25] and WG [26] and, although our observations need to be confirmed in larger cohorts, FHL and secondary HLH are also candidate diseases.

In accord with the pathophysiological processes described above, the chronic low-level activation of the Th1 system in our patient (moderately elevated steady-state levels of IP-10 and IL-18BP, but not of TNFα) may result from the persistent stimulation delivered by the host's own, perforin-deficient cells. Interestingly, a similar mechanism appears to be involved in the pathogenesis of influenza caused by the H5N1 virus, as the H5 hemagglutinin suppresses perforin expression [30] which essentially results in an acquired form of HLH, including dysregulation of cytokines [31], [32]. Furthermore, some viruses encode secretable IL-18BP which interferes with the host's immune response by reducing free IL-18. For example, the lesions caused by molluscum contagiosum virus are nearly void of inflammatory cells [33].

An association of IL-8 with HLH has been described for the secondary [34], but not the familial disease. Neutrophils are not considered a first-line problem in FHL; however, pathological neutrophil behavior can occur in related clinical situations [35], [36]. As IL-8, which was markedly elevated in our patient, aggravates inflammation by recruiting neutrophils to sites of active disease, our data indicate that the role of neutrophils in HLH may be underappreciated.

Presently, there is no link of IL-27 to HLH. This cytokine can promote a Th1 response in its early stages [37] and thus may contribute to disease progression in FHL. On the other hand, the predominant function of IL-27 is anti-inflammatory (e.g., inhibition of Th2, Th17, and late-stage Th1 responses, as well as of macrophages and NK cells [38]), suggesting that the upregulation of IL-27 may constitute one of the negative feedback mechanisms which are commonly initiated in response to inflammation. It is possible that IL-27 is partially effective in reducing the severity of the disease in the patient, achieving a healthy phenotype, but failing to restore an entirely normal status of the immune system.

Our data are obtained from a single patient and her parents; thus, any conclusion needs to be drawn with reasonable caution. We anticipate that this limitation may encourage future research to corroborate our findings and elaborate on several aspects, especially on a possible therapeutic role of IL-18BP in HLH. Summarizing our data, we suggest that assessing IL-18BP, IP-10, and/or IL-8 may assist in identifying latent disease activity in transplanted FHL patients whose clinical condition and laboratory status is still normal. IL-8 and IL-27 are introduced as possible new players in FHL. Furthermore, different degrees of abnormalities of cytokine regulation in the heterozygous parents shed light on genotype-phenotype correlations in FHL. Most importantly, we assert that the capacity of IL-18BP to counterbalance excessive production of Th1 cytokines was defective in the patient. This may constitute a novel mechanism of disease perpetuation which, given the pathogenetic similarities, may also be relevant in SLE, WG, and H5N1 infections.

## References

[1] Farquhar JW, Claireaux AE (1952). Familial haemophagocytic reticulosis.. Arch Dis Child.

[2] Caballes RL, Caballes-Ponce MG, Kim DU (1997). Familial hemophagocytic lymphohistiocytosis (FHLH).. Pathology.

[3] Stepp SE, Dufourcq-Lagelouse R, Le Deist F, Bhawan S, Certain S (1999). Perforin gene defects in familial hemophagocytic lymphohistiocytosis.. Science.

[4] Busiello R, Adriani M, Locatelli F, Galgani M, Fimiani G (2004). Atypical features of familial hemophagocytic lymphohistiocytosis.. Blood.

[5] Muralitharan S, Wali YA, Dennison D, Lamki ZA, Zachariah M (2007). Novel spectrum of perforin gene mutations in familial hemophagocytic lymphohistiocytosis in ethnic Omani patients.. Am J Hematol.

[6] Cote M, Menager MM, Burgess A, Mahlaoui N, Picard C (2009). Munc18-2 deficiency causes familial hemophagocytic lymphohistiocytosis type 5 and impairs cytotoxic granule exocytosis in patient NK cells.. J Clin Invest.

[7] Henter JI, Elinder G, Soder O, Hansson M, Andersson B (1991). Hypercytokinemia in familial hemophagocytic lymphohistiocytosis.. Blood.

[8] Ishii E, Ohga S, Aoki T, Yamada S, Sako M (1991). Prognosis of children with virus-associated hemophagocytic syndrome and malignant histiocytosis: correlation with levels of serum interleukin-1 and tumor necrosis factor.. Acta Haematol.

[9] Osugi Y, Hara J, Tagawa S, Takai K, Hosoi G (1997). Cytokine production regulating Th1 and Th2 cytokines in hemophagocytic lymphohistiocytosis.. Blood.

[10] Dinarello CA (2007). Interleukin-18 and the pathogenesis of inflammatory diseases.. Semin Nephrol.

[11] Mazodier K, Marin V, Novick D, Farnarier C, Robitail S (2005). Severe imbalance of IL-18/IL-18BP in patients with secondary hemophagocytic syndrome.. Blood.

[12] Takada H, Nomura A, Ohga S, Hara T (2001). Interleukin-18 in hemophagocytic lymphohistiocytosis.. Leuk Lymphoma.

[13] Takada H, Ohga S, Mizuno Y, Suminoe A, Matsuzaki A (1999). Oversecretion of IL-18 in haemophagocytic lymphohistiocytosis: a novel marker of disease activity.. Br J Haematol.

[14] Jordan MB, Hildeman D, Kappler J, Marrack P (2004). An animal model of hemophagocytic lymphohistiocytosis (HLH): CD8+ T cells and interferon gamma are essential for the disorder.. Blood.

[15] Novick D, Kim SH, Fantuzzi G, Reznikov LL, Dinarello CA (1999). Interleukin-18 binding protein: a novel modulator of the Th1 cytokine response.. Immunity.

[16] Henter JI, Arico M, Egeler RM, Elinder G, Favara BE (1997). HLH-94: a treatment protocol for hemophagocytic lymphohistiocytosis. HLH study Group of the Histiocyte Society.. Med Pediatr Oncol.

[17] Henter JI, Samuelsson-Horne A, Arico M, Egeler RM, Elinder G (2002). Treatment of hemophagocytic lymphohistiocytosis with HLH-94 immunochemotherapy and bone marrow transplantation.. Blood.

[18] Lehrnbecher T, Bernig T, Hanisch M, Koehl U, Behl M (2005). Common genetic variants in the interleukin-6 and chitotriosidase genes are associated with the risk for serious infection in children undergoing therapy for acute myeloid leukemia.. Leukemia.

[19] Lehrnbecher T, Chanock SJ (2003). Detection of common cytokine and colony stimulating factor gene polymorphisms.. Methods Mol Biol.

[20] Fishman D, Faulds G, Jeffery R, Mohamed-Ali V, Yudkin JS (1998). The effect of novel polymorphisms in the interleukin-6 (IL-6) gene on IL-6 transcription and plasma IL-6 levels, and an association with systemic-onset juvenile chronic arthritis.. J Clin Invest.

[21] Paulukat J, Bosmann M, Nold M, Garkisch S, Kampfer H (2001). Expression and release of IL-18 binding protein in response to IFN-gamma.. J Immunol.

[22] Kaser A, Novick D, Rubinstein M, Siegmund B, Enrich B (2002). Interferon-alpha induces interleukin-18 binding protein in chronic hepatitis C patients.. Clin Exp Immunol.

[23] Hurgin V, Novick D, Rubinstein M (2002). The promoter of IL-18 binding protein: activation by an IFN-gamma -induced complex of IFN regulatory factor 1 and CCAAT/enhancer binding protein beta.. Proc Natl Acad Sci U S A.

[24] Bachmann M, Paulukat J, Pfeilschifter J, Muhl H (2008). Molecular mechanisms of IL-18BP regulation in DLD-1 cells: pivotal direct action of the STAT1/GAS axis on the promoter level.. J Cell Mol Med.

[25] Novick D, Elbirt D, Miller G, Dinarello CA, Rubinstein M (2009). High circulating levels of free interleukin-18 in patients with active SLE in the presence of elevated levels of interleukin-18 binding protein.. J Autoimmun.

[26] Novick D, Elbirt D, Dinarello CA, Rubinstein M, Sthoeger ZM (2009). Interleukin-18 binding protein in the sera of patients with Wegener's granulomatosis.. J Clin Immunol.

[27] Kogawa K, Lee SM, Villanueva J, Marmer D, Sumegi J (2002). Perforin expression in cytotoxic lymphocytes from patients with hemophagocytic lymphohistiocytosis and their family members.. Blood.

[28] Sullivan KE, Delaat CA, Douglas SD, Filipovich AH (1998). Defective natural killer cell function in patients with hemophagocytic lymphohistiocytosis and in first degree relatives.. Pediatr Res.

[29] Katano H, Cohen JI (2005). Perforin and lymphohistiocytic proliferative disorders.. Br J Haematol.

[30] Hsieh SM, Chang SC (2006). Insufficient perforin expression in CD8+ T cells in response to hemagglutinin from avian influenza (H5N1) virus.. J Immunol.

[31] Cheung CY, Poon LL, Lau AS, Luk W, Lau YL (2002). Induction of proinflammatory cytokines in human macrophages by influenza A (H5N1) viruses: a mechanism for the unusual severity of human disease?. Lancet.

[32] Lipatov AS, Andreansky S, Webby RJ, Hulse DJ, Rehg JE (2005). Pathogenesis of Hong Kong H5N1 influenza virus NS gene reassortants in mice: the role of cytokines and B- and T-cell responses.. J Gen Virol.

[33] Smith VP, Bryant NA, Alcami A (2000). Ectromelia, vaccinia and cowpox viruses encode secreted interleukin-18-binding proteins.. J Gen Virol.

[34] Tamura K, Kanazawa T, Tsukada S, Kobayashi T, Kawamura M (2008). Increased serum monocyte chemoattractant protein-1, macrophage inflammatory protein-1beta, and interleukin-8 concentrations in hemophagocytic lymphohistiocytosis.. Pediatr Blood Cancer.

[35] Kfoury Baz EM, Mikati AR, Kanj NA (2002). Reactive hemophagocytic syndrome associated with thrombotic thrombocytopenic purpura during therapeutic plasma exchange.. Ther Apher.

[36] Kono T, Takigawa M, Nishimura F, Takashiba S, Nakagawa M (1997). Host defensive, immunological, and microbiological observations of an early-onset periodontitis patient with virus-associated hemophagocytic syndrome.. J Periodontol.

[37] Takeda A, Hamano S, Yamanaka A, Hanada T, Ishibashi T (2003). Cutting edge: role of IL-27/WSX-1 signaling for induction of T-bet through activation of STAT1 during initial Th1 commitment.. J Immunol.

[38] Batten M, Ghilardi N (2007). The biology and therapeutic potential of interleukin 27.. J Mol Med.

